# Multi-method Analysis of Implementation Determinants and Strategies to Support the Implementation of Contingency Management for Methamphetamine Use in an HIV Care Setting

**DOI:** 10.1007/s10461-026-05115-4

**Published:** 2026-05-13

**Authors:** Maximo R. Prescott, Victoria Chentsova, Jorge Delgado, Devin Dung, Steven Shoptaw, Gregory A. Aarons, David J. Moore, David Grelotti, Jessica Montoya

**Affiliations:** 1 HIV Neurobehavioral Research Program, University of California San Diego, 220 Dickinson St # B, San Diego, CA 92103 USA; 2 San Diego State University/University of California San Diego Joint Doctoral Program in Clinical Psychology, San Diego, CA USA; 3 Department of Family Medicine, University of California, Los Angeles, Los Angeles, CA USA; 4 Department of Psychiatry, University of California San Diego, La Jolla, San Diego, CA USA

**Keywords:** HIV, Contingency management, Methamphetamine use, HIV primary care, Implementation determinants, Implementation strategies, Implementation science

## Abstract

Methamphetamine use is prevalent among people with HIV (PWH) and complicates HIV care. Integrated substance use and HIV care services are recommended, yet rarely available. Contingency management (CM) is an evidence-based treatment for methamphetamine use, but its implementation within HIV primary care is limited. This study identified determinants and strategies to support CM implementation in HIV care. Key informants from an HIV clinic at an academic medical center (*n* = 15 patients; *n* = 15 providers) completed semi-structured interviews and a survey. A rapid qualitative analysis guided by the Exploration, Preparation, Implementation, Sustainment (EPIS) framework identified implementation determinants, and potential strategies were classified using the Expert Recommendations for Implementing Change (ERIC) compilation. Both patient and provider participants rated CM as acceptable (*M* = 3.72 and 4.55/5) and appropriate (*M* = 3.85 and 4.54/5). Providers also rated CM as feasible (*M* = 4.08/5) and reported strong support for integrating CM into HIV care, citing the high priority of addressing methamphetamine use. Facilitators included the clinic’s person-centered culture and providers’ familiarity with substance use treatment. Barriers included perceptions that reinforcer values in standard CM protocols were low, limited staffing, and competing provider demands. Suggested implementation strategies included adapting CM (e.g., increasing reinforcer value), patient-directed outreach/advertisements, and provider education and support (e.g., reminders about CM availability and referral processes). Findings indicate strong support for integrating CM into HIV primary care, while noting challenges to implementation. Addressing these determinants through targeted multi-level implementation strategies may enhance CM adoption and sustainment in HIV care settings.

## Resumen

El uso de metanfetamina es frecuente entre las personas con VIH (PCV) y complica su tratamiento. Aunque se recomiendan servicios integrados para el tratamiento de sustancias y el VIH, rara vez están disponibles. La gestión de contingencias (CM) es un tratamiento con evidencia para la metanfetamina, pero su implementación en la atención primaria del VIH es limitada. Este estudio identificó determinantes y estrategias para apoyar la implementación de CM en entornos de atención del VIH. Informantes clave de una clínica de VIH en un centro médico académico (*n* = 15 pacientes; *n* = 15 proveedores) completaron entrevistas semiestructuradas y una encuesta cuantitativa. Un análisis cualitativo rápido guiado por el marco Exploration, Preparation, Implementation, Sustainment (EPIS) identificó determinantes de la implementación, y las estrategias se clasificaron utilizando la compilación Expert Recommendations for Implementing Change (ERIC). Pacientes y proveedores calificaron la CM como aceptable (M = 3.72 y 4.55/5) y apropiado (M = 3.85 y 4.54/5); los proveedores también lo consideraron factible (M = 4.08/5) y apoyaron su integración en la atención del VIH, citando la alta prioridad de abordar el consumo de metanfetamina. Los facilitadores incluyeron la cultura clínica centrada en la persona y la experiencia previa de los proveedores en tratamiento de adicciones. Las barreras principales incluyeron el bajo valor percibido de los incentivos en los protocolos estándar de CM, la escasez de personal y las demandas concurrentes de los proveedores. Se propusieron estrategias como adaptar la CM (p. ej., aumentar los incentivos), divulgación dirigida a pacientes (p. ej., anuncios publicitarios) y formación y apoyo a proveedores (p. ej., recordatorios sobre la CM y aclaración de procesos de derivación). Los resultados muestran fuerte apoyo para integrar la CM en la atención primaria del VIH, aunque existen desafíos de implementación. Abordar estos factores determinantes mediante estrategias de implementación dirigidas y multinivel puede favorecer la adopción y sostenibilidad de la CM en entornos de atención del VIH.

## Introduction

Methamphetamine use is hampering efforts to end the HIV epidemic (EHE). Reaching the public health benefits of antiretroviral therapy (ART) through treatment as prevention (TasP), which requires sustained adherence to ART to reduce onward HIV transmission rates [1], is greatly undermined by prevalent comorbidities, including substance use disorders (SUDs) [2]. Although efforts in the United States are justifiably focused on combating the opioid epidemic, methamphetamine use represents a persistent and growing national concern [3]. People with HIV (PWH) use methamphetamine at higher rates than the general population [4] and methamphetamine use is linked to persistent gaps in the HIV care continuum: a greater risk of HIV seroconversion [5], delayed HIV testing for diagnosis [6], failure to link to HIV care [7], difficulties being retained in HIV care [8, 9], and an increased likelihood of having a detectable HIV viral load [10, 11]. Methamphetamine use has also been alarmingly linked to a greater risk of mortality among PWH [12]. Even without complete abstinence from methamphetamine, reductions in use yield meaningful benefits for closing gaps in the HIV care continuum [13]. Among PWH who use meth, engagement in SUD treatment is associated with 3-fold greater odds of being adherent to ART [8]. Thus, there is an urgent need to adapt and implement evidence-based treatments for methamphetamine use, particularly in clinical settings serving PWH.

Current clinical practice guidelines from the American Society of Addiction Medicine and American Academy of Addiction Psychiatry identify contingency management (CM) as the standard of care for stimulant use disorder (StUD), including methamphetamine use disorder (MUD), because it has demonstrated the best effectiveness of any studied treatment [14]. These guidelines further recommend CM as the primary component of treatment plans, while noting that it can be combined alongside other evidence-based behavioral therapies (e.g., CBT, Matrix Model) and/or off-label pharmacotherapies (e.g., bupropion, naltrexone, mirtazapine), particularly given the absence of any FDA-approved medications for StUD. As a behavioral therapy based on operant conditioning, CM decreases the reinforcing effects of substance use by providing immediate positive reinforcement (e.g., vouchers or drawings to earn prizes) following biological evidence of abstinence from the substance (e.g., negative urine toxicology) [15, 16]. CM has been shown to be an effective intervention for reducing methamphetamine use among those seeking substance use treatment [17–19]. Despite initial perceptions that CM may be cost-prohibitive, healthcare systems (e.g., the Department of Veterans Affairs) and states (e.g., California) have launched implementation initiatives to expand patients’ access to CM, particularly for patients meeting criteria for StUD [20–23]. Furthermore, research has also found CM for MUD to be cost-effective, including in real-world settings [18, 24].

Although CM is among the most effective behavioral interventions for methamphetamine use, its dissemination and implementation remain limited despite the recognized need and benefits of integrated SUD treatment for PWH [25]. CM has demonstrated strong efficacy in reducing methamphetamine use and improving treatment retention, yet its adoption is hindered by barriers including lack of funding, limited provider training, and organizational constraints such as staffing and competing clinical priorities in other real-world settings [26–29]. Existing research has also indicated that provider perspectives play a central role in the implementation of CM. Across clinical settings, providers commonly report concerns related to funding and reimbursement, legal and regulatory uncertainty, training and fidelity demands, and stigma or philosophical discomfort with incentives, all of which can hinder CM adoption and sustainment [27, 29]. Moreover, when CM is implemented, fidelity and competency to evidence-based protocols may be low and undermine its impact. To address these challenges, structured implementation strategies have been proposed such as the Science to Service Laboratory (SSL) model, which combines didactic training, performance feedback, and external facilitation [30, 31]. This multi-level approach is designed to support organizational readiness, improve CM delivery fidelity, and enhance sustainment across opioid treatment programs.

Importantly, CM also has a robust evidence base among PWH, which has primarily been used to target stimulant use and/or ART adherence. Multiple trials among PWH have demonstrated the efficacy of CM for reducing stimulant use, improving treatment retention, and achieving viral suppression [32–39]. While several studies have successfully delivered CM for methamphetamine in community-based and clinical settings serving PWH [39, 40], it remains rarely integrated into routine HIV care settings despite its strong efficacy and higher rates of methamphetamine use among PWH. HIV care settings may also differ in important ways from many of the clinical contexts in which implementation determinants and strategies for CM have previously been studied, including provider perspectives on the intervention. HIV providers typically deliver long-term, relationship-based care and manage complex multimorbidity, including high rates of co-occurring SUDs, mental health conditions, and social instability [41, 42]. In one study examining CM for alcohol within HIV care settings, provider perspectives were also influenced by HIV-specific priorities such as retention in care, viral suppression, and the acceptability of interventions that target behavior change without increasing stigma [43]. These clinical realities may uniquely shape how HIV providers evaluate the acceptability, appropriateness, and feasibility of implementing CM in their clinical settings. However, there is limited empirical work examining the multilevel implementation determinants, such as organizational context, provider workflows, and intervention fit, as well as the strategies needed to support CM adoption and sustainment within HIV care settings. Addressing this gap is essential for translating CM from a research-supported intervention into routine clinical practice for PWH using methamphetamine.

The present study aimed to examine patient and provider participants’ perspectives on implementation barriers, facilitators, and strategies for delivering CM for methamphetamine use in a busy HIV care setting.

## Methods

### Setting

Study participants (both patients and providers) were recruited from an academic medical center’s HIV primary care clinic in San Diego, CA. This clinic is the largest HIV primary care clinic in San Diego and one of the largest HIV clinics in the U.S., serving more than 3,000 PWH. Furthermore, the clinic population closely resembles the demographics of San Diego County’s HIV epidemic: it is predominantly male (88%), has a mean age of 53 years, is racially and ethnically diverse (52% White, 33% Hispanic), with nearly half (46%) identifying as lesbian or gay, and the prevalence of methamphetamine use has been estimated to be 17% [44]. This study and its associated study procedures were reviewed and approved by the University of California San Diego’s Institutional Review Board.

### Participants

A total of thirty participants were recruited and interviewed from the HIV primary care clinic of an academic medical center, which included both providers and patients. Fifteen of those participants were clinic patients with moderate to high risk of MUD and had a willingness to share their thoughts about SUD services. The other fifteen were providers/staff (e.g., infectious disease specialists, internists, licensed vocational nurses, behavioral health providers) willing to share their perspectives about SUD services. All participants in this study were 18 years or older. Few exclusion criteria were applied to enhance the generalizability of the sample.

### Implementation Science Frameworks

The present study was guided by the Exploration, Preparation, Implementation, and Sustainment (EPIS) framework, which serves both as a process model outlining four key stages of implementation and as a determinant framework identifying influential factors, such as characteristics of the intervention and bridging factors (i.e., dynamic interactions between outer and inner organizational contexts), that influence implementation outcomes [45, 46]. This study primarily aligns with the EPIS “Preparation” phase, focusing on assessing contextual factors influencing CM implementation and identifying potential strategies to support its adoption within an HIV primary care clinic where CM for methamphetamine was not routinely available to patients. Within the EPIS framework, the inner context was considered to be the HIV primary care clinic, while the outer context encompassed broader influences such as the academic medical system and state and federal policies and funding levels [47]. Innovation factors were conceptualized as features of CM that could impact its implementation and uptake. The primary bridging factor, or linkages between the inner and outer contexts, was conceptualized as the academic research partnership involved in the conduct of the study.

Additionally, the Expert Recommendations for Implementing Change (ERIC) compilation informed the analysis and classification of implementation strategies recommended by participants. ERIC includes 73 discrete strategies identified via a modified Delphi process involving implementation experts [48], which have since been categorized into nine conceptual domains [49]. Utilizing the ERIC taxonomy enabled us to apply standardized terminology when describing types of strategies proposed to support CM implementation within an HIV primary care setting. This structured approach facilitates the design of contextually tailored multicomponent implementation strategies aimed at integrating evidence-based practices (EBPs) like CM into routine practice.

### Data Collection

In this multi-method study, data were collected through two different methods: a brief web-based survey and key informant interviews. The brief survey was designed to be completed in under 15 min, and informant interviews were generally 30 to 60 min. All enrolled participants completed the web-based survey regarding their sociodemographic characteristics (age, sex, gender identity, race/ethnicity, and sexual orientation), as well as their impression of CM potentially being offered at their clinic (measures described below). Additionally, research team members conducted semi-structured interviews regarding implementation of CM with key informants using an interview guide informed by the EPIS framework. The interview guide also included a brief description and explanation of CM, as well as questions exploring implementation strategies (including adaptations) needed for the program to better fit patient needs within an HIV primary care clinic. Specifically, participants were presented with a standardized description of a twelve-week, voucher-based contingency management protocol featuring escalating monetary reinforcement contingent on methamphetamine-negative urine screens. The protocol began at $2 for the first negative screen and increased with consecutive abstinent samples, with resets following positive or missed samples. A rapid-reset feature was also described, whereby participants who produced three consecutive methamphetamine-negative urine samples following a reset would return to the reinforcer value they had earned prior to the positive or missed sample. This protocol was purposely chosen to reflect a CM model for methamphetamine commonly implemented in real-world clinical settings [18, 50].

### Measures

*Acceptability*,* Appropriateness*,* and Feasibility.* The Acceptability of Intervention Measures (AIM), Intervention Appropriateness Measure (IAM), and Feasibility of Intervention Measure (FIM) were used to gauge the acceptability, appropriateness, and feasibility of the intervention, respectively [51]. Each is a 4-item scale, with response options on a scale from 1 to 5, with a higher average score being considered more acceptable, appropriate, or feasible for intervention.

*Contingency Management Beliefs.* The Contingency Management Beliefs Questionnaire (CMBQ) measures healthcare providers’ attitudes and support of the CM program [52]. It is a 32-item assessment, and its values range on a scale from 1 to 5 with a higher average score being considered a strong belief in the program.

*Implementation Intentions.* The Measure of Innovation-Specific Implementation Intentions (MISII) assesses providers’ willingness to adopt and use new practices [53]. It is a 3-item assessment, and its values range on a scale of 1 to 5 with a higher average score signaling a higher willingness to adopt these practices.

*Substance Use.* The Alcohol, Smoking, and Substance Involvement Screening Test (ASSIST) is used to identify individuals that are at risk of problematic substance use [54]. Each substance is scored individually with responses being summed for each to produce a total risk score, such that higher scores are indicative of higher risk of problems associated with substance use and substance dependence. The instrument was adapted to replace amphetamines more specifically with methamphetamine given the specific aims of the study.

### Data Analysis

Given the focused research questions of the present study, qualitative data were analyzed following a rapid qualitative approach [55, 56], which has been found to enhance efficiency while preserving the rigor and validity of traditional qualitative methods [57–59]. Interviews were transcribed, verified for accuracy, and de-identified prior to analysis. The Planning for and Assessing Rigor in Rapid Qualitative Analysis (PARRQA) framework was further used to inform the rigorous design, data collection, summary template development, matrix analysis, and rapid qualitative data synthesis of the project [60]. A standardized transcript summary guide was developed for rapid analysis, structured around a-priori domains derived from the interview guide and the guiding EPIS framework. These domains captured relevant factors from the inner and outer settings, as well as innovation characteristics that could influence implementation. Four research team members piloted the transcript summary guide, after which it was consistently applied to all transcripts. For each summary, one team member completed the initial summary, which was subsequently audited by a second team member for accuracy. Disagreements in summaries were first resolved by discussion between the two analysts, while a third analyst was involved if consensus could not be reached. After summaries had been completed, they were organized into a matrix table, aligning with the a-priori domains, to enable systematic comparison and thematic synthesis across participants [55, 56]. Guided by the EPIS framework and the ERIC strategies compilation, two analysts independently reviewed the matrix to identify key implementation determinants and implementation strategies for CM. To ensure validity, the summaries and emerging themes were regularly reviewed together by the larger analysis team.

For the quantitative survey data, descriptive statistics (i.e., mean, median, standard deviations, interquartile range, percentages) were calculated to characterize the provider and patient participant samples. Given the relatively small sample sizes of participants, inferential statistics were not conducted.

## Results

### Participant Characteristics

#### Provider Participants

Fifteen provider participants, characterized as any individual providing care at a participating HIV clinic, participated in an in-depth interview followed by a brief survey. Provider participant characteristics are detailed in Table 1. Provider participants were predominantly male (60.0%), white (53.3%), and had a professional degree (e.g., MD, DO, PhD, 46.7%). Official roles of providers varied but mostly consisted of physicians, nurses, and social workers, in addition to a physician assistant, clinical pharmacist, behavioral health counselor, and medical director. Across roles, provider participants had an average of 6.43 years (SD = 6.07) of experience at their present clinic.

**Table 1:** Provider participant characteristics

Characteristics	
Age (Median [IQR])	44.0 [38.5, 51.5]
Sex	N (%)
Male	9 (60.0%)
Female	6 (40.0%)
Gender	
Male	9 (60.0%)
Female	6 (40.0%)
Race and ethnicity	
White ^NH^	8 (53.3%)
Black ^NH^	1 (6.7%)
Asian American ^NH^	4 (26.7%)
Hispanic or Latino	2 (13.3%)
Education	
Completed some college	2 (13.3%)
Completed Master’s Degree (e.g., MS, MPH, MSN)	6 (40.0%)
Completed professional or Graduate degree (e.g., MD, PhD)	7 (46.7%)

^NH^ = Non-Hispanic

#### Patient Participants

Fifteen patient participants participated in an in-depth interview and completed a brief survey. Patient participant characteristics are detailed in Table 2. Participants had a mean age of 50.0 years old and predominantly identified as male (93.3%), gay or lesbian (53.3%), white (46.7%) or Hispanic/Latino (46.7%), with some college education (40.0%). Most reported being currently unemployed (86.6%) with about half currently on disability (40.0%). All patient participants reported having insurance, including private health insurance (either through their employer or an Affordable Care Act plan), California Medicaid, or some other public coverage including safety net insurance for PWH. In considering connectivity, all patient participants reported owning a cell phone and having it on a regular basis. Two of these patient participants, however, reported at least one cell phone number change over the past year, and two reported their cell number being out of service at least once over the past year.

**Table 2:** Patient participant characteristics

Characteristics	
Age (Median [IQR])	50.0 [39.5, 53.5]
Sex (N (%))	
Male	14 (93.3%)
Female	1 (6.7%)
Gender	
Male	14 (93.3%)
Female	1 (6.7%)
Sexual orientation	
Straight or heterosexual	3 (20.0%)
Gay or Lesbian	8 (53.3%)
Bisexual	1 (6.7%)
Queer, pansexual, and/or questioning	2 (13.3%)
*Missing*	1 (6.7%)
Race and ethnicity	
White ^NH^	7 (46.7%)
American Indian or Alaskan Native ^NH^	1 (6.7%)
Hispanic or Latino	7 (46.7%)
Education	
Graduated high school or obtained GED	4 (26.7%)
Some college	6 (40.0%)
Graduated college (bachelor’s degree)	2 (13.3%)
Graduate degree	3 (20.0%)
Employment Status	
Yes, Full-time	2 (13.3%)
No, currently unemployed	5 (33.3%)
No, currently on disability	6 (40.0%)
No, other	2 (13.3%)
Insurance type	
Private health insurance or HMO	2 (13.3%)
California medicaid	9 (60.0%)
Affordable care act plan	2 (13.3%)
Safety net insurance for PWH (Ryan White)	1 (6.7%)
Other public insurance	1 (6.7%)
ART Adherence (Mean (SD))	90.4 (12.1)
*Missing*	1 (6.7%)
Injection of Amphetamines	
No, never	4 (26.7%)
Yes, but not in the past 3 months	3 (20.0%)
Yes, in the past 3 months	5 (33.3%)
*Missing*	3 (20.0%)
ASSIST (Mean (SD))	
Methamphetamine	19.0 (8.91)
* Missing*	5 (33.3%)
Alcohol	10.0 (9.59)
* Missing*	6 (40.0%)
Cannabis	11.0 (6.63)
* Missing*	7 (46.7%)

The mean ASSIST risk score for methamphetamine was 18.9 for participants, which falls within the moderate risk range. Most patient participants endorsed some injection methamphetamine use historically (53.3%), with about a third (33.3%) endorsing injection use in the last three months. Polysubstance use was uncommon across patient participant self-report. Of those reporting polysubstance use, average ASSIST scores indicated lower dependence risk with alcohol, moderate dependence risk with cannabis, moderate dependence risk with cocaine, high dependence risk with opioids, and moderate dependence risk across other substances (unspecified).

### Acceptability, Appropriateness, and Feasibility

On the quantitative measures, provider participants reported higher ratings of acceptability and appropriateness of CM than patient participants. Across provider participants, the acceptability of CM was rated at an average of 4.55 of 5.0, slightly higher than the average rating across patient participants at 3.72. Similarly, across provider participants, appropriateness of CM was rated at an average of 4.54 of 5.0, while patient participants rated appropriateness at an average of 3.85. Finally, regarding the feasibility of CM, provider participants rated it an average of 4.08 of 5. The summary of CM ratings across provider and patient participants (where applicable) can be found in Table 3.

**Table 3:** Acceptability, appropriateness, feasibility of contingency management

	Provider participants	Patient participants
Acceptability (AIM) [Mean (Standard Deviation)]	4.55 (0.544)	3.72 (1.15)
Appropriateness (IAM)	4.54 (0.611)	3.85 (0.940)
*Missing*	*1 (6.7%)*	
Feasibility (FIM)	4.08 (0.572)	N/A
CM beliefs questionnaire (CMBQ)		N/A
General barriers	0.784 (0.518)	
*Missing*	3 (20.0%)	
Training barriers	1.07 (0.684)	
Supportive statements	3.13 (0.765)	
Plans to Refer to CM [N (%)]		N/A
Not at all	0 (0.0%)	
To a slight extent	0 (0.0%)	
To a moderate extent	3 (20.0%)	
To a great extent	6 (40.0%)	
To a very great extent	6 (40.0%)	
Priority of referring to CM		N/A
Not at all	0 (0.0%)	
To a slight extent	2 (13.3%)	
To a moderate extent	1 (6.7%)	
To a great extent	6 (40.0%)	
To a very great extent	6 (40.0%)	

### Qualitative Findings

Provider and patient participants identified several implementation determinants, which included facilitators and barriers to implementing CM for methamphetamine in an HIV primary care clinic setting. These implementation determinants were characterized using the EPIS framework and are presented in Table 4.

**Table 4:** Implementation determinants of contingency management for methamphetamine use in a HIV care setting coded by the EPIS framework

EPIS		Theme	Provider participants	Patient participants
Domain	Construct			
Inner context	Individual characteristics	Provider knowledge of SUD treatment or referrals	“If a patient is highly motivated and engaged, I would usually refer them to my colleagues who specialize in medication-assisted treatment and also to any open psychologists and psychiatrist if they're available at the time. In the circumstance where someone is not quite sure and they have a good relationship with me and they don't seem—and they have reluctance for additional visits, that's the setting that I usually would provide MATs for my patients.”	“...my HIV doctor... she's trying to do more with addiction, in helping out with addiction, That would be my primary. I would like her to know, see if it's working...” “Well, there's my number one person would be my healthcare provider...I've just found out that if you just be honest with them, they're not really going to judge me... Then they can finally diagnose me and say, okay, this is what we use today.”
		Competing provider demands	“...we are a teaching facility that there's so many other projects that might get mixed with something else...” “I think there’s just so much on the plate of providers in a clinic session... we are partly the patient’s primary care specialist, but also their medical specialist or HIV specialist...also supporting patients who are unstably housed, who have other social service needs, food access... There’s just a lot in a 30-minute visit to then also remember to refer patients.”	
	Organizational characteristics	SUD as a treatment priority	“...we would solve so many problems in our clinic and our community if we had as effective substance use treatment strategies...so I would put that as number one... it would be like the Lazarus effect, like when they started highly active antiretroviral therapy.”	
		Interdisciplinary collaboration	“...it's getting more robust all the time...we have psychiatry. We have medically assisted treatment. We have two substance use counselors. We have social workers, some of whom are very specialized in substance use. We have a psychologist who is providing groups. Those are all available to them, and I wanna make sure they know that, especially in the beginning.”	
		Person-centered culture	“I would say, yeah, overall, the clinic, we all follow harm reduction model. Everyone's accepted, no matter what's going on in their life."	“It's a systematic change that has to happen with the doctors and everybody. You guys are the pioneers. You're doing this by helping out. You are doing a great job of being honest and being forthcoming. ‘We're trying to help you. We're not judging you.’”
		Behavioral health staffing	"Things get pushed out a little bit. That I've noticed especially for new referrals. There's two and a half months...Some of the patients lose interest after a while. I think that's kind of what lacks sometimes, is the availability, should I say." “More staffing, more personnel, more professional personnel, mental health providers, need more of that...” "I think, also, if we had behavioral health counselors...we only have two, so they’re always pretty booked up...if we can have them be available to talk to them at that moment, more like immediate access, I think that would be nice also."	
Outer context	Patient/client characteristics	Prevalence of methamphetamine use	“Meth is king here so – and queen and everything else.” “I think our patients are particularly vulnerable.. . [substance use] can come up, literally, with every single patient.”	“The opioids were great, but it just wasn’t doing it for me as the crystal meth was, so what ended up happening was I went ahead and just fell back into that as well just to get that initial high ’cause it just wasn’t enough for me, so I needed more and more and more.” “I did start to build my life around trying to obtain it [meth] any way that I could... I moved down to <City>, <Country>... it’s at every corner there. It was easy to understand how to get it there.”
		Willingness and readiness for SUD treatment	“…you can’t instill the desire [for treatment] in the patient. If their desire has passed, I think the more you try to force them to do something against their will, they begin to resent it. I think that goes against what maybe the contingency management idea is trying to evoke.” “Some people are afraid to try something new because change is scary.”	“It’s good, if you can do it, if you have that mindset. It’s all in the mindset. If you have that mindset, you have that drive and that if you want it, here’s a way to do it.”
		SUD and engagement in HIV care	“It impacts every step of their care, and we know that people who use drugs are not doing as well along the care continuum as people who do not.” “Some of the patients... they forget to take their meds, they forget to take care of themselves. Half the time they forget that they have to make their way here...”	“It's routine for me. At this stage I may have missed one or two this whole past year, I would say. Other than that I just don't miss it; I just don't. It's part of my routine. It's like coffee, breakfast, substance, and then my medication.” “ If I was high the day before [an HIV care appointment] I would wait maybe a day and just push it back. I wouldn't get high on the day if I knew I was going to go get it.” “Well, I wasn’t taking any medications when I was using… It was just not on my radar.”
		Social determinants of health	“...substance use and then mental health concerns and housing are probably the three biggest barriers to our patients getting the care that they need and deserve.” “They tend to be LGBT or drug users or somehow on—somehow 'othered' in society.” “... people who use have—often have disorganized lifestyles, may be homeless. It's hard to take medications regularly if other things are higher priority.”	“... the problem with drug use usually stems from a lot deeper places than you need some money, so having financial incentives really isn't that big in the long run, because people who use drugs, they probably don't have a house. People who use drugs probably don't have family. People who use drugs probably have childhood trauma, unresolved issues that they'd never talked about. I think that's what continues to drive people's drug use. Not going to the root of the problem and trying kill the weed from the top, that's not gonna work...”
		Nature of addiction		“The nature of addiction would be another. All sorts of motivations have been tried with me, and I still lost two jobs. I still was estranged from my kids…” “You need to find out exactly where that person's stem...A lot of people really don't understand what they're getting themselves into until they're in it.”
	Funding/contracting	Financial sustainment	“Well, funding is the biggest thing... ensuring that even despite the sometimes limited insurance that our population have, that the role of psychologist is able to be funded appropriately, whether that's through ongoing contribution from the clinic or not.” “There are a host of other barriers. I think it's not at the provider level. It's at the health system level, the administrative level. I think the administration is a little inflexible. They just don't know how to manage these types of things. They've never done it before.”	
Innovation factor	Innovation/ EBP fit Innovation/ EBP characteristics	Evidence-Based Practice	“I think we’ve seen studies in different areas that use this type of positive reinforcement that have had good outcomes.”	
		Positive reinforcement	“I think that the positive reinforcement idea is certainly great.”	“The ability to have to stay clean. It’s more forceful, in a way. You could look at it like, ‘You have to do it this way. It can’t be positive.’ Just more of the rough reinforcement, I think would be good.” “I think it's great incentive. I think money's great. I think I've been through some other—I think I've been receiving the negative incentive from schizophrenia over here, that I think some positive money encouragement, I think I can do. I think I'm all down for it.”
		Patient fit	"I think it just relates to being very patient-centered, meeting patients where they’re at." “I think it's needed, and it's pretty specific to the population we deal with for sure just based on the chronically homeless and the substance use.” “I think it doesn't—on the surface, it doesn't look like it creates or contributes to a sense of judgment or shame around substance use. That, I think, is appealing.” “The problem I see with twice a week is actually getting them to show up twice a week.”	" Maybe the inconvenience of having to go twice a week to do a sample, but that’s how you’re gonna do it. That’s how you quit. That’s how you succeed in the program. Those would be the only things that I see other people see as an inconvenience.” “I just want it to be known that everybody's treatment is going to be drastically different from the next person, so to cookie-cutter a huge plan for millions of people struggling with addiction to me is not sensical...They need to be very individualized.”
		Incentive salience	“I'm not sure that $2 would be a very choice prize. That's not a lot. Costs are going up everywhere.” “I hate to patronize my patients, and some of these interventions can come across as a little bit patronizing… I think it’s just trying to find the right patients to do this for, to provide it.”	“...it’s a very powerful addiction, and holding that carrot—that’s not a big carrot just to hold in front of somebody who’s a real addict.”
		Broader impacts	"Yeah, so definitely decreased methamphetamine use, also any increased adherence to their medical appointments, increased adherence to their HIV medications, any reduction in other risk behaviors...and then just an increase in quality-of-life indicators.”	
		Accountability		“...It's nice to get an incentive, but I’m not necessarily doing it for that. I wanna do it for accountability. To me, it works. I don't want—I'm very competitive. I'm going to have every single test be negative. That makes me accountable... because I wanna make sure that it's negative tests that I'm going to. I'm completely accountable for that.”

#### Inner Context: HIV Primary Care Clinic

Provider participants highlighted multiple characteristics of providers and their organizational environment that could serve to facilitate, or act as barriers to, implementation of CM in an HIV primary care setting at the level of the inner context. The high feasibility of CM was facilitated by providers’ pre-existing knowledge of SUD treatments and referrals, as well as the organization’s interdisciplinary, person-centered culture and the high priority of treating SUD within the HIV care setting, while the competing provider demands and a perceived shortage of behavioral health staffing were identified as potential barriers.

##### Individual Characteristics

Provider participants, regardless of specialty, consistently reported on their interest and knowledge of SUDs, enabling them to make appropriate referrals or provide services directly. As one provider participant reported:

*Provider participant: “I would usually refer them to my colleagues… in the circumstances where someone is not quite sure and they have a good relationship with me… I usually would provide medications for addiction treatment [MAT] for my patients.”*

Patient participants shared a similar sentiment. Many identified their HIV care providers for support during CM, given their familiarity with addiction. Such *provider knowledge of SUD treatments and referrals* may provide essential channels facilitating CM referrals.

Provider participants, however, also highlighted the challenges of *competing provider demands*. They noted that as providers not primarily focused on SUDs, other health concerns may take priority during visits, limiting the time available to discuss SUDs. Furthermore, these competing demands also limit the time providers have to learn about new treatment modalities or referral opportunities. Altogether, competing demands may serve as barriers to both providers’ ability to be informed about CM and to address substance use substantially enough to make a referral to CM within a single visit.

*Provider participant: “I think there’s just so much on the plate of providers in a clinic session… there’s just a lot in a 30-minute visit to then also remember to refer patients.”*

##### Organizational Characteristics

On the organizational level, provider participants reported *SUD as a treatment priority* for its impact on HIV-related health behaviors and outcomes. Provider participants also reflected on how *interdisciplinary collaboration* is foundational to the feasibility of providing substance use treatment in their clinic. Many reported working in teams of different specialists across the HIV care continuum, from social work to behavioral health to psychiatry, providing robust care for SUD among general health concerns. Furthermore, provider and patient participants alike reflected on the *patient-centered culture* of their organizations, highlighting a common harm-reductionist orientation taken in providing substance use related care.

*Provider participant: “everyone’s accepted, no matter what’s going on in their life.”*

Further, patient participants highlighted the importance of this non-judgmental and accepting environment providers curate in facilitating patient empowerment to pursue treatment for SUD. As one patient participant stated:

*Patient participant: “It’s a systematic change that has to happen with the doctors and everybody. You guys are the pioneers. You’re doing this by helping out. You are doing a great job of being honest and being forthcoming. ‘We’re trying to help you. We’re not judging you.’”*.

Despite a supportive culture and SUD as a treatment priority, provider participants also identified challenges in *behavioral health staffin*g leading to delays and loss to follow-up in provision of specialized substance use care, which may act as a barrier to implementing CM. As one provider participant stated:

*Provider participant: “More staffing*,* more personnel*,* more professional personnel*,* mental health providers*,* need more of that.”*

#### Outer Context: Broader Community

Provider and patient participants also highlighted multiple characteristics of the outer context including the patient population and the broader organizational context. Across the broader context, provider and patient participants consistently highlighted similar implementation determinants of CM in an HIV care setting.

##### Patient Characteristics

Across interviews, provider participants consistently reported high prevalence of methamphetamine use and related concerns among other SUDs.

*Provider participant: “I think our patients are particularly vulnerable… [substance use] can come up*,* literally*,* with every single patient.”*

Patient participant interviews revealed a consistent sentiment, with many highlighting the relative availability of methamphetamine over other drugs. As one patient participant stated:

*Patient participant: “I did start to build my life around trying to obtain it [meth] any way that I could… I moved down to… it’s at every corner there. It was easy to understand how to get it there.”*

Such prevalence of methamphetamine use highlights not only the appropriateness but the necessity for interventions targeting methamphetamine use within HIV care settings.

Relatedly, provider participants highlighted that methamphetamine use frequently interferes with *engagement in care*, from failing to show up to appointments, to having difficulties keeping up with medication responsibilities outside of the clinic setting. As one provider participant put it:

*Provider participant: “Some of the patients… they forget to take their meds*,* they forget to take care of themselves. Half the time they forget that they have to make their way here.”*

Interestingly, patient participant perspectives on the impact of methamphetamine use and engagement with HIV Care were relatively more mixed compared to providers. Some patient participants claimed that adherence to antiretroviral medications and following appointment schedules is routine to them, with some even scheduling their methamphetamine use around those activities. Others shared the opposite experiences, suggesting everything from pushing back HIV appointments to accommodate their methamphetamine use schedules, to complete disregard for antiretroviral medications. One patient participant echoed the provider participant’s concerns:

*Provider participant: “Well*,* I wasn’t taking any medications when I was using… it was just not on my radar.”*

Provider participants also highlighted the challenge of patients having varying *readiness and willingness to engage* in substance use treatment for methamphetamine. While this may prove to facilitate referrals or and/or engagement in CM for motivated patients, it may also serve as a barrier to many patients who are not interested in treatment. Provider participants also highlighted that not only may this serve as a barrier in the short term, but that putting pressure on patients to engage in treatments like CM may contribute to long-term resentment and misalignment between patients and providers.

*Provider participant: “…you can’t instill the desire [for treatment] in the patient. If their desire has passed*,* I think the more you try to force them to do something against their will*,* they begin to resent it. I think that goes against what maybe the contingency management idea is trying to evoke.”*

Similarly, patient participants highlighted that capitalizing on willingness and readiness for treatment are key facilitators to successful engagement in treatment.

*Patient participant: “It’s all in the mindset. If you have that mindset*,* you have that drive*,* and that is you want it*,* here’s a way to do it.”*

Further contributing to challenges in engagement and willingness, provider participants also spoke on the common presence and impact of comorbidities and *adverse social determinants of health*, including but not limited to other mental health concerns, stigma and discrimination, financial instability, and housing status. Provider participants called particular attention to how these adverse social determinants of health could both serve as a barrier to meaningful engagement in CM (e.g., not having reliable transportation to attend frequent appointments) and would not be directly treated by CM, falling short of the facets of addiction that many patients would like addressed. Patient participant commentary was in line with the provider participants, speaking to the importance of recognizing how social determinants of health play into the addictive cycle and how treatments like CM may not address it.

*Patient participant: “…the problem with drug use usually stems from a lot deeper places because people who use drugs*,* they probably don’t have a house. People who use drugs probably don’t have family. People who use drugs probably have childhood trauma*,* unresolved issues that they’d never talked about. I think that’s what continues to drive people’s drug use. Not going to the root of the problem and trying kill the weed from the top*,* that’s not gonna work…”*.

Relatedly, patient participants described the *nature of addiction* as heterogenous and multifaceted, complicating the feasibility of any treatment and may act as a barrier to its application as it fails to capture the nuance of methamphetamine use.

*Patient participant: “You need to find out exactly where that person’s [methamphetamine use] stem… A lot of people really don’t understand what they’re getting themselves into until they’re in it.”*

##### Funding/Contracting

Beyond patient characteristics, provider participants also spoke to challenges in financial sustainment of offering new treatment programs and the need to engage with health system leadership to demonstrate a strong financial case for providing funding for new programming. Though provider participants themselves are not frequently responsible for securing program funding, many spoke to the challenges they hear at the administrative level that could serve as a barrier to sustaining a program like CM in HIV care settings.

*Provider participant: “There are a host of other barriers. I think it’s not at the provider level. It’s at the health system level*,* the administrative level. I think the administration is a little inflexible. They just don’t know how to manage these types of things. They’ve never done it before.”*

#### Innovation Factors: CM for Meth

Finally, provider and patient participants both highlighted specific characteristics of CM that may facilitate or serve as a barrier to implementation of CM in an HIV primary care setting.

##### EBP Fit / EBP Characteristics

Though CM was new to most provider participants, many found it appealing for the potential of broader impact on health behaviors as a potential facilitator to making referrals. Specifically, provider participants expressed hope that, beyond reducing methamphetamine use, CM could also improve appointment engagement, medication adherence, and other health-related risks.

Provider participants also highlighted CM’s status as an evidence-based practice as contributing to its acceptability. Some provider participants specifically highlighted that research demonstrating utility of CM facilitates their interest in referring patients to it. Relatedly, many provider participants further commented on the innovative positive-reinforcement focused approach of CM as supporting its acceptability and implementation in an HIV care setting. As one provider participant stated:

*Provider participant: “I think we’ve seen studies in different areas that use this type of positive reinforcement that have had good outcomes.”*

Patient participants similarly found the positive-reinforcement aspect of CM to be appealing, especially when so many had been met with approaches seemingly focusing on negative reinforcement.

*Patient participant: “I think it’s great incentive… I think I’ve been receiving the negative incentive from schizophrenia over here*,* that I think some positive money encouragement*,* I think I can do. I think I’m all down for it.”*

Although, both provider and patient participants appreciated the positive reinforcement aspect of CM, both also raised concerns about **incentive salience** of the proposed reinforcer schedule. More specifically, both provider and patient participants raised concerns that low reinforcer values may serve as a barrier to implementation:

*Patient participant: “…it’s a very powerful addiction*,* and holding that carrot—that’s not a big carrot just to hold in front of somebody who’s a real addict.”*

*Provider participant: “I’m not sure that $2 would be a very choice prize. That’s not a lot. Costs are going up everywhere.”*

In addition to the positive reinforcement, some patient participants spoke to the accountability provided by CM as a facilitator. Specifically, patient participants reported that, while positive reinforcement is greatly appreciated, the frequency of visits would provide the necessary accountability many patients need to initiate their recovery journey.

*Patient participant: “…It’s nice to get an incentive*,* but I’m not necessarily doing it for that. I wanna do it for accountability…I’m very competitive. I’m going to have every single test be negative. That makes me accountable…”*.

Despite the interest in EBPs from some provider participants, accountability from some patient participants, and shared excitement for the positive reinforcement approach, almost all provider and patient participants interviewed also spoke to the importance of considering individual characteristics of patients and overall patient fit in considering the appropriateness of CM.

*Patient participant: “I just want it to be known that everybody’s treatment is going to be drastically different from the next person*,* so to cookie-cutter a huge plan for millions of people struggling with addiction to me is not sensical…They need to be very individualized.”*

Some provider participants suggested the potential fit of CM for patients struggling with finances. However, provider and patient participants also brought up concerns about the time and travel requirements which may be barriers to participation for patients experiencing financial hardship.

#### Implementation Strategies: Engaging Providers and Patients

Provider and patient participants identified several implementation strategies to support adoption of CM for methamphetamine in HIV primary care settings in the context of these implementation determinants. These implementation strategies were characterized using the ERIC framework and are presented in Table 5.

**Table 5:** Participant identified implementation strategies of contingency management for methamphetamine use in a HIV care setting coded by the ERIC framework

ERIC		Theme	Provider participant quote	Patient participant quote
Category	Strategy			
Support clinicians	Facilitate relay of clinical data to providers	Motivating referrals with outcome data	“Also, I think if the results or the progress so far could be shared… I think if the providers can see that sending these patients to this program is beneficial … would be helpful just to keep the providers engaged.”	
	Remind clinicians	Reminding providers	“I think maybe we just need periodic reminders that the [CM] study is still going on, that the resources are still available… over time, it kind of gets forgotten because there are other competing studies that are asking for attention as well…”	
Train and educate stakeholders	Conduct educational meetings	Provider/staff education	“… and I think it’s the lack of education… if providers have more of an understanding of what contingency groups really are, especially if they’re not part of social services, psychiatry, and stuff… I think that would be helpful”	“That team has to remind you and give you—definitely give you encouragement and has to be able to be—your doctors should be able to talk to you… I think that, and the doctors themselves need to be aware of these studies. They have to encourage you to do this [CM], saying, this is a huge benefit that you have.”
	Distribute educational materials	Provider-facing educational materials	“Just making sure there’s buy-in from all the providers and that, yeah, you’ve got your literature in their rooms so that referrals are easy.”	
Engage consumers	Use mass media	Advertising directly to patients	“Certainly having posters and signage in the waiting rooms…then also mailers, mailing information out to patient physically…they could certainly make advertisements at the center in < City>, make some connections with < Organization > or some of the rehab sources around town that we’re often referring patients to, educating them about this community resource as well.”	“If there would be some way you guys could join up with the needle exchange, I think that might be some way…If you guys can get ‘em into the clinic, and then you guys can incentivize them.” “You have to be part of the community—excuse me. Those would be big stomping points.” “A lotta people are very secretive about their usage, or don’t like to talk about it, so I really couldn’t say unless you put up flyers in the waiting room…”
	Prepare patients/consumers to be active participants	Patient initiated referrals	“… send direct MyChart messages to patients who might be eligible. It takes the provider out of it a little bit, but obviously, the message would have to be that this is something that you might be eligible for. Speak to your provider about this if you’d be interested.” “I think the flyers and all that stuff, I think it’s always great direct patient marketing, so if patients ask us about it, then I think it gets done. It’s hard for us to always remember all the things we have…”	“Maybe, having advocates. I’m willing to pick up flyers the next time I’m in there and take them up to < Location > or wherever I go out to… or just in the car and pass them out.” “Reaching out to them [patients] and just pretty much being aware of it and then just pretty much brought up in doctors’ visits and stuff… usually that or through MyChart as well.”
Adapt and tailor to context	Tailor strategies	Streamlined referral process	“If it [referrals] was something easy to do, that would be the easiest thing to do. If you created a dot phrase that we could put on the after-visit study…something along those lines just to increase the ease of how referrals would go.”	
		Comprehensive and integrated care	“We have resources that will refer patients to, and perhaps a patient is stabilized, and then the provider, the primary care provider, takes over routine maintenance or management… It embeds contingency management into a lifestyle, an overall behavioral lifestyle change, as opposed to something that’s performed twice a week for a certain team of providers in the clinic.”	“Probably ‘cause you’re not offering a package… Here’s money for staying sober. Okay, I can do that, but what about therapy?. Something where maybe I could call somebody if I’m feeling down… Maybe something hand in hand with that, like also supportive services, which I’m sure I can find somewhere else, but you guys are offering here why not offer it as a package?” “If they see a patient fit, just like Dr. < Name > did. He recommended me for this, because he thought I was a good fit for the program. Really, knowing your patients and where you can put them.”
	Promote adaptability	Accessibility adaptations	“All that being said I think taking into account those limitations I think it could serve those folks that would have difficulties in being able to come to the clinic twice a week…psychologists, telehealth has been used in psychiatry and psychology for a very long time, so I know that they can do it.”	
		Adapt reinforcement schedule	“I think a lot of patients are not gonna want to come in twice a week…I do think a lot of patients will want to come into clinic and have capacity to come into the clinic. For those folks, it’s gonna be great to… be flexible in how it’s offered… We really gonna want your second urine to be negative, but we’ll give you money to come to your first contingency management orientation.”	“You want to pay? Pay for this. That would be appealing to me. Pay for my phone. Pay for my internet. Let me have a little more breathing room with my financials. That would be appealing.” “It would be nice if it were more, but anything is better than nothing.”
Utilize financial strategies	Access new funding	Diversifying sustainable funding sources	“At some point, it may be worthwhile to even try to seek a donor, and maybe someone who’s willing to help sustain a program like that… having to apply for money and grants all the time is not easy, but I think at some point, maybe trying to move towards finding a donor long term that can provide support that way might be an interesting direction to look at.”	

##### Support Clinicians: Bringing CM To The Forefront

Given the competing demands and shortage of behavioral health staff, the need to support clinicians came up consistently across provider participant interviews. Specifically, provider participants reported the need to *Facilitate Relay of Clinical Data to Providers* in the form of updates on patient outcomes and engagement with CM that would motivate providers to refer their patients to CM. Provider participants also highlighted the importance of *Reminding Clinicians* regarding the availability of CM programming, which may help reduce the burden of keeping track of active treatment programming and ensure referrals can be followed through on. One provider participant highlighted:

*Provider participant: “I think maybe we just need periodic reminders…that the resources are still available…over time*,* it kind of gets forgotten.”*

##### Train and Educate Stakeholders: Informing Catalysts of CM

Relatedly, as many HIV care staff facilitate patient engagement in substance use treatment, both provider and patient participants commented on the importance of having clinic staff be well-educated on CM. Understanding of CM may help streamline referrals and have open conversations with patients. Many provider participants highlighted the need for training and education on CM for hospital staff to better equip them to make referrals to CM.

*Patient participant: “…your doctors should be able to talk to you… I think that*,* and the doctors themselves need to be aware of these studies. They have to encourage you to do this [CM]*,* saying*,* this is a huge benefit that you have.”*

Some provider participants suggested *Conducting Educational Meetings* could provide a formal space to develop a better understanding of CM. These meetings could be used to both provide education and even specialized training for interdisciplinary staff within the clinic. Other provider participants also suggested the importance of *Distributing Educational Materials*, which could be readily available in examination rooms for both providers and patients to support the implementation and adoption of CM. Together, these strategies were proposed to improve buy-in from providers and support more referrals to CM.

##### Engage Consumers: Empowering Patients

In addition to strategies to facilitate provider referrals to CM, provider and patient participants recommended directly engaging with patients with CM advertising to support adoption. Both provider and patient participants shared various strategies, from having providers ready to relay CM information when requested to utilizing patient champions of CM to spread awareness, all to *Prepare Patients to Be Active Participants* and initiate their own referrals.

*Provider participant: “They [patients] definitely ask their providers if there are any studies [CM] they’re eligible for…”*.

*Patient participant: “Maybe*,* having advocates. I’m willing to pick up flyers the next time I’m in there and take them up to < Location > or wherever I go out to… or just in the car and pass them out.”*

Relatedly, most provider and patient participants provided some suggestions related to *Using Mass Media* to advertise CM directly to patients and mitigate reliance on providers to provide referrals. The channels through which to use mass media recommended varied greatly, reflecting the heterogeneity of individuals likely to benefit from CM. Furthermore, with varying degrees of comfort in public discussion of methamphetamine use treatment, varying forms of advertisement become necessary.

*Patient participant: “A lotta people are very secretive about their usage*,* or don’t like to talk about it*,* so I really couldn’t say unless you put up flyers in the waiting room…”*.

This could include engaging with physical media, such as signage in waiting rooms and public spaces throughout the city, particularly spaces serving communities known to be at high risk of HIV (e.g., syringe exchanges, bathhouses). This could also include engaging with virtual media such as dating applications, social networking site communities, or sending patients advertisements through appointment management platforms such as MyChart. Both groups, but especially patient participants also spoke to the importance of working with other organizations (e.g., rehabilitation programs) to promote the availability of CM. As one patient participant stated:

You have to be part of the community—excuse me. Those would be big stomping points.

##### Adapt and Tailor to Context: Recognizing Patient Needs

Provider participants emphasized the need to tailor implementation strategies to address clinic-level barriers to CM—such as behavioral health staff shortages and competing demands—by developing efficient, streamlined referral processes and leveraging the clinic’s interdisciplinary structure to support referrals across disciplines. For example, several provider participants suggested developing a “dot phrase” in their electronic health record to add to the after-visit notes to facilitate referrals to CM.

*Provider participant: “If it [referrals] was something easy to do*,* that would be the easiest thing to do.”*

While many provider and patient participants highlighted the appropriateness of CM, many suggested further adaptations to CM to ensure patient fit, particularly for patients affected by adverse social determinants of health. Specifically, they recommended integrating CM into a comprehensive and integrated care package, preferably in-house, to address interconnected health behaviors. Provider participants especially emphasized the utility of embedding CM in a more comprehensive portfolio of care.

*Provider participant: “It embeds contingency management into a lifestyle*,* an overall behavioral lifestyle change*,* as opposed to something that’s performed twice a week for a certain team of providers in the clinic.”*

Patient participants further emphasized that, to facilitate this comprehensive and integrated care, it is critical for providers to understand the full needs of their patients. As one patient participant stated:

*Patient participant: “If they see a patient fit*,* just like Dr. < Name > did. He recommended me for this*,* because he thought I was a good fit for the program. Really*,* knowing your patients and where you can put them.”*

Provider and patient participants also highlighted various strategies to *Promote Adaptability*. Provider participants spoke of the importance of accessibility adaptations, such as telehealth, ensuring the benefits of intervention could be available to the largest group of people while acknowledging individual needs. Both patient and provider participants also highlighted the need to adapt the reinforcement schedule to be more engaging, particularly in the face of the demands of CM programming. One provider participant, for instance, suggested offering reinforcers to individuals for attending their weekly appointments may be a helpful adaptation. Many patient and provider participants agreed the reinforcer value may not be enough to support meaningful engagement with the program. Patient participants also suggested adapting the reinforcement mode to be more flexible than the proposed gift cards, so it could be used to pay bills and more meaningful impact social determinants of health (e.g., housing):

*Patient participants: “You want to pay? Pay for this. That would be appealing to me. Pay for my phone. Pay for my internet. Let me have a little more breathing room with my financials. That would be appealing.”*

##### Utilize Financial Strategies: Building Program Sustainment

In response to concerns around the financial sustainment of CM, multiple provider participants suggested *Access to New Funding* in the interest of diversifying funding sources (e.g., public grants, private donors) to support the sustainment and long-term accessibility of CM programming.

## Discussion

### Principal Findings

This study identified key implementation determinants and strategies to support the adoption and sustainment of CM for methamphetamine use within an HIV primary care setting. Both patient and provider participants perceived CM as an acceptable and appropriate intervention, while providers also believed it was feasible for their clinical setting if appropriate funding could be provided. Within the inner context, the clinic’s strong person-centered culture, providers’ extensive knowledge of SUD treatment, and established interdisciplinary collaboration emerged as important implementation facilitators. At the level of the outer context, methamphetamine use was perceived as a top priority in HIV care due to its high prevalence and detrimental impact on treatment engagement and outcomes. Patient fit emerged as an important determinant, as both patients and providers described readiness or willingness to engage in treatment for methamphetamine as shaping referral decisions and potential engagement within HIV primary care.

Regarding the innovation itself, CM was viewed positively for its emphasis on positive reinforcement, accountability, and its strong evidence base. However, several barriers were also identified across contexts, including competing provider demands and limited behavioral health staffing, the influence of social determinants of health, concerns about financial sustainment of CM, and insufficient incentive salience (low reinforcer value) in existing CM protocols. To address these barriers, stakeholders suggested implementation strategies such as adapting CM to the clinic context (e.g., increasing or diversifying reinforcers, packaging with other interventions), providing provider training and ongoing reminders to support referrals, engaging patients as active participants through direct-to-consumer advertising, and leveraging new funding mechanisms to ensure financial sustainment.

### Comparisons with Prior Work

Consistent with prior findings on the implementation determinants of CM, our findings also suggest that successful implementation of CM is shaped by organizational culture, provider attitudes and capacity, funding sustainment, and access to training and support for providers during implementation [26–29]. However, our study importantly extends this literature in the context of addressing methamphetamine use in HIV care settings, as prior studies of CM have largely focused on community substance use treatment or primary care settings [27] despite the high prevalence (48%) of SUDs [61]. In doing so, we identified several unique determinants, including strong alignment between CM and the clinic’s person-centered culture. While Ledgerwood et al. identified stigma and negative staff attitudes toward substance use and/or CM as prominent implementation barriers [59], we found that clinic culture, providers’ familiarity with SUD treatment, and positive attitudes toward CM emerged as implementation facilitators, suggesting the relative appropriateness and feasibility of CM for methamphetamine within HIV care settings. Additionally, although efficacy trials of contingency management have intentionally recruited non–treatment-seeking populations and nonetheless demonstrated robust effects on substance use [62–64], our findings suggest that in routine HIV care settings, perceived willingness and readiness to engage with substance use treatment may still function as an implementation determinant shaping who is ultimately offered or referred to CM.

 Previous studies have also found that concerns about financial sustainment of CM are likely to hamper its adoption outside of research settings with grant funding, in large part due to historical regulatory restrictions prohibiting offering reinforcers to patients [26, 27, 29]. However, the legal and policy environment is beginning to shift, as of March 2025, eight states had applied for Sect. 1115 Demonstration Waivers to pilot CM programs for SUD treatment, with five already approved (California, Washington, Montana, Hawaii, and Delaware), indicating growing federal and state support for CM implementation beyond research contexts [65]. In California, where this study was conducted, the Recovery Incentives Program represents the first large-scale implementation of CM funded by Medicaid, offering up to $599 in reinforcers over six months to support stimulant abstinence [23]. Ensuring that providers are aware of emerging changes in federal and state initiatives and continuing to advocate for publicly insured funding mechanisms is critical to overcoming a persistent implementation barrier and improving CM adoption and sustainment. As our findings suggest that the value of the reinforcer matters, it will be important to evaluate if $599 over six months is found to be meaningful for people with active methamphetamine use. Importantly, perspectives on incentive magnitude in the present study must be interpreted in light of the specific CM protocol described to participants. Participants were presented with a voucher-based CM model beginning at a $2 reinforcer value, which is substantially lower than incentive magnitudes recommended in recent evidence-based guidance that accounts for inflation [66]. As such, the participant and provider recommendations to increase reinforcer values in our study likely reflect recognition that the specific described protocol may be insufficiently reinforcing, rather than dissatisfaction with contingency management as an intervention more broadly. This interpretation is consistent with the extensive CM literature demonstrating that larger incentive magnitude is a key determinant of treatment effectiveness [67]. At the same time, increasing incentive salience may introduce additional implementation challenges, especially funding, sustainment, and policy constraints, which warrant careful evaluation in future implementation studies.

In addition to accessing new funding, provider and patient participants also identified the importance of multi-level implementation strategies to generate referrals to CM for methamphetamine use through provider training, education, support and direct-to-patient advertising. Previous implementations and rollouts of CM in the United States have similarly stressed the importance of addressing limited provider capacity through efficient training and support programs. Notably, the Veterans Health Administration conducted the largest nationwide implementation of CM to date, supporting over 100 clinics through a combination of centralized funding for CM reinforcers, structured regional trainings, and ongoing expert consultation to ensure fidelity and provider engagement [21]. Becker and colleagues further demonstrated that one-time trainings alone are insufficient and that multi-level implementation models such as the Science to Service Laboratory (SSL), which combine counselor-level strategies (e.g., behavioral rehearsal, performance feedback) with organizational-level supports (e.g., external facilitation, leadership engagement, and workflow integration), can substantially improve and sustain CM adoption in real-world community treatment settings [68]. While prior large-scale efforts such as the VA rollout and the SSL model have focused primarily on provider- and organizational-level strategies to expand CM, key informants in our study uniquely emphasized the value of direct-to-consumer advertising to engage patients as active stakeholders. Both patient and provider participants noted that such advertising could raise awareness of CM, empower patients to initiate discussions about evidence-based care, and facilitate self-referrals—extending multi-level implementation efforts to include patients as drivers of adoption of CM.

### Limitations and Strengths

This study has some limitations. The sample was drawn from a single, well-resourced HIV primary care clinic at an academic medical center, which may limit generalizability to other clinics with fewer resources or different organizational structures. For example, geographic variation in substance use patterns among PWH, such as higher rates of methamphetamine use in the Western United States and greater cocaine use in the Northeast, may further constrain the generalizability of our findings regarding CM for methamphetamine to regions where different substances predominate [69]. Thus, studying the implementation determinants and strategies of CM for methamphetamine within other HIV care settings, including less resourced and non-academic settings, is an important future direction to determine the extent these findings generalize. Additionally, not all staff roles were equally represented among provider participants, in part due to scheduling constraints and union-related challenges (e.g., being unable to participate in research activities not within the purview of their job description during working hours) that made research participation more difficult for some clinic personnel. The study’s cross-sectional design captures perceptions during the preparation phase of implementation, but longitudinal data is needed to assess how determinants and strategies evolve over time and across implementation stages. Moreover, while the sample size was appropriate for qualitative inquiry [70], the small sample size limited the extent to which quantitative comparisons could be made. Because of this limited quantitative power, we were also unable to conduct mixed-methods integration procedures that would formally merge qualitative and quantitative data; however, the quantitative data were still useful for descriptive sample characterization, and some convergence across methods was evident given the overlapping constructs assessed. Finally, our concurrent elicitation of determinants and strategies within the same session may have shaped how participants generated strategies; future longitudinal studies could address this by separating these processes and following intervention mapping procedures to more systematically link determinants to tailored strategies [71].

The study also has several notable strengths. It represents one of the first efforts to examine the implementation of CM for methamphetamine within an HIV primary care context, offering novel findings into how this evidence-based intervention can be best implemented within HIV primary care settings. Additionally, we followed best-practice guidelines for rapid qualitative analysis to ensure the methodological rigor and validity of our findings [60]. While most implementation determinants were jointly identified by both patient and provider participants, the inclusion of multiple perspectives enhanced the depth of our findings as there were several determinants uniquely identified by each group given their complementary perspectives. Finally, our use of the EPIS implementation framework and ERIC compilation to guide data collection and analysis are a notable strength that foster the interpretability and external validity of our findings to best support future implementation of CMs in other healthcare settings, especially HIV care.

## Conclusion

This study identified key multilevel determinants and strategies to support the implementation of CM for methamphetamine in an HIV primary care setting. Patient and provider participants viewed CM as acceptable, appropriate, and feasible, underscoring the strong alignment between CM’s principles and the person-centered culture of HIV care. However, barriers such as limited behavioral health staffing, low incentive salience, and financial sustainment challenges must be addressed to support adoption and sustainment. Implementing targeted, multilevel strategies—including provider training and reminders, patient engagement initiatives, and sustainable funding mechanisms—may enhance CM uptake and long-term viability. These findings provide a foundation for developing scalable, contextually tailored implementation strategies to expand access to evidence-based treatments for methamphetamine within HIV care settings.
